# Exploring Metabolic Pathway Reconstruction and Genome-Wide Expression Profiling in *Lactobacillus reuteri* to Define Functional Probiotic Features

**DOI:** 10.1371/journal.pone.0018783

**Published:** 2011-04-29

**Authors:** Delphine M. Saulnier, Filipe Santos, Stefan Roos, Toni-Ann Mistretta, Jennifer K. Spinler, Douwe Molenaar, Bas Teusink, James Versalovic

**Affiliations:** 1 Department of Pathology and Immunology, Baylor College of Medicine, Houston, Texas, United States of America; 2 Department of Translational Biology and Molecular Medicine Program, Baylor College of Medicine, Houston, Texas, United States of America; 3 Center for Integrative Bioinformatics, Vrije Universiteit Amsterdam, Amsterdam, The Netherlands; 4 Kluyver Centre for Genomics of Industrial Fermentation, Netherlands Consortium for Systems Biology, Delft, The Netherlands; 5 Department of Pathology, Texas Children's Hospital, Houston, Texas, United States of America; 6 Department of Microbiology, Swedish University of Agricultural Sciences, Uppsala, Sweden; University of North Carolina at Charlotte, United States of America

## Abstract

The genomes of four *Lactobacillus reuteri* strains isolated from human breast milk and the gastrointestinal tract have been recently sequenced as part of the Human Microbiome Project. Preliminary genome comparisons suggested that these strains belong to two different clades, previously shown to differ with respect to antimicrobial production, biofilm formation, and immunomodulation. To explain possible mechanisms of survival in the host and probiosis, we completed a detailed genomic comparison of two breast milk–derived isolates representative of each group: an established probiotic strain (*L. reuteri* ATCC 55730) and a strain with promising probiotic features (*L. reuteri* ATCC PTA 6475). Transcriptomes of *L. reuteri* strains in different growth phases were monitored using strain-specific microarrays, and compared using a pan-metabolic model representing all known metabolic reactions present in these strains. Both strains contained candidate genes involved in the survival and persistence in the gut such as mucus-binding proteins and enzymes scavenging reactive oxygen species. A large operon predicted to encode the synthesis of an exopolysaccharide was identified in strain 55730. Both strains were predicted to produce health-promoting factors, including antimicrobial agents and vitamins (folate, vitamin B_12_). Additionally, a complete pathway for thiamine biosynthesis was predicted in strain 55730 for the first time in this species. Candidate genes responsible for immunomodulatory properties of each strain were identified by transcriptomic comparisons. The production of bioactive metabolites by human-derived probiotics may be predicted using metabolic modeling and transcriptomics. Such strategies may facilitate selection and optimization of probiotics for health promotion, disease prevention and amelioration.

## Introduction

Human beings are colonized by a diverse and complex collection of microbes, contributing to host nutrition, development of the immune system, response to pathogens, and intestinal cell differentiation and proliferation [1], [2]. A global initiative represented by the International Human Microbiome Consortium (IHMC) is currently characterizing microbial communities that reside in diverse body habitats, and how the human microbiome may contribute to health and disease [1]. By 2012, this consortium aims to sequence 1000 bacterial genomes to serve as reference strains and help researchers to better understand the evolutionary relationships within the microbiome. As part of the Human Microbiome Project (HMP), the genomes of four human-derived *Lactobacillus reuteri* strains (*L. reuteri* ATCC PTA 6475, ATCC PTA 4659, ATCC 55730, and CF48-3A) have been sequenced [1]. *L. reuteri* is a bacterium indigenous to humans and other animals, and has been isolated from infants and adults at different body sites, including the gastrointestinal (GI) tract, vagina, and from human breast milk [3], [4].

Host adaptation of this species is supported by genetic clustering of strains originating from common or related hosts [5]. Using amplified-fragment length polymorphism (AFLP) and multi-locus sequence analysis (MLSA) with more than 160 *L. reuteri* strains isolated from humans (including the 4 strains sequenced as part of the HMP), pigs, rats, mice, chickens and turkeys revealed that considerable genetic heterogeneity exists within the *L. reuteri* population, with distinct monophyletic clades reflecting host origins [6]. Preliminary genomic analysis from four human-derived *L. reuteri* strains sequenced by the HMP suggested that these strains can be divided into two different groups (strains 55730 and CF48-3A in one group, and strains 6475 and 4659 in a separate group and highly similar to the type strain JCM 1112) [1]. These findings confirm previous observations from Oh *et al* showing that at least two different clades of human-derived *L. reuteri* strains can be distinguished, as represented in the phylogenetic tree created using amplified fragment length polymorphism analysis [6].

*L. reuteri* 55730 is an established probiotic strain, defined as “bacterium, which when ingested in an adequate amount, confer a health benefit to the host” [7]. The beneficial effects of oral intake of *L. reuteri* strain ATCC 55730, or its daughter strain DSM 17938 [8], have been well documented in a number of clinical trials. These strains improved symptoms of infantile colic [9], feeding tolerance and gut function in pre-term infants [10], and reduced constipation or perceived pain intensity in children with functional abdominal pain [11]. Treatment with strain 55730 also modulated cytokine patterns in exhaled breath condensates of children with atopic dermatitis [12]. *L. reuteri* ATCC PTA 6475 is a promising probiotic strain with anti-inflammatory properties demonstrated *in vitro* [13]. This strain has been shown to ameliorate disease due to enterohemorrhagic *Escherichia coli* in germ-free mice [14]. Most human-derived *L. reuteri* strains secreted the antimicrobial aldehyde reuterin [6] that is active against a wide range of pathogens [15]. Human-derived *L. reuteri* strains from two different groups differed by their immunomodulatory properties, ability to produce the antimicrobial factor reuterin, and form biofilms *in vitro* [13], [16], [17], [18]. However, within each of these two groups, the strains are highly similar to one another [1], [5].

Probiogenomics was the term recently coined to define whole genome sequencing of probiotics [19] as a strategy to generate insights into the functional diversity, health-promoting mechanisms, and evolution of probiotics. This approach has been used in a relatively small number of probiotics that belong mostly to the *Lactobacillus* and *Bifidobacterium* genera [20], [21], [22]. Although complete genome sequences describe all known metabolic reactions for a specific bacterial strain, genomics does not address quantitative differences in gene expression and how different genes and metabolic pathways are interconnected. Transcriptome analysis effectively complements microbial genomics by enabling one to map gene expression profiles onto metabolic models established by genomic DNA sequencing data. Microbial transcriptomics may yield improved predictions of metabolic processes and enzymatic pathways present in well-defined environmental conditions, but interpretations of such metabolic models are cumbersome [23], [24]. Visualization and interpretation of transcriptomics data is facilitated by the use of genome-scale metabolic networks for individual bacterial strains [25], but the generation of pan-metabolic networks for entire bacterial species and metabolic pathway reconstruction tools for microbial communities remain largely under development. In this study, we focused on genome-wide comparisons of two human breast milk-derived *L. reuteri* strains, including one established commercial strain (*L. reuteri* ATCC 55730), with distinct genetic and functional features in an attempt to explain host survival and probiotic mechanisms. Metabolic models were created for both strains *L. reuteri* 55730 and 6475, and these models highlighted different probiotic features in each strain. The transcriptomes of each strain were visualized and compared in different growth phases in order to explore potential physiologic differences in the host. A recent study by Van Baarlen et al [26] highlighted the functional differences in human subjects following administration of a single *L. plantarum* strain harvested in two different growth phases. The metabolic modeling approach in this study revealed potential differences of functional significance between strains of a single probiotic species and enabled metabolic pathway prediction that could be useful for the optimization of survival, persistence and efficacy of probiotic strains. Although few metabolic pathways were found to be unique to each strain, genomic comparisons revealed extensive differences between these strains due to large genomic rearrangements in 55730, and the presence of large numbers of genes encoding proteins of unknown function in both *L. reuteri* strains 6475 and 55730. Transcriptome comparisons revealed many differences between these strains when grown in a semi-defined medium, providing a rational basis for selection of strains for different human applications, improved commercial formulations, and nutritional insights that could enhance strain effectiveness *in vivo*.

## Results

### Genome-Wide Comparisons

The genome sequences of strains 55730 and 6475 are relatively similar in size. The genome of strain 6475 is composed of a single circular chromosome of 2,039 kb [5], while the genome of strain 55730 is composed of a single chromosome together with 4 plasmids (2,036 kb). The sequences of these plasmids have been described previously and named as pLR580, pLR581, pLR584, pLR585, ranging in size from 8.1 to 19.1 kb [8]. Plasmids pLR581and pLR585 contain tetracycline [*tet*(W)] and lincosamide resistance genes [*lnu*(A)], respectively, have been successively removed from *L. reuteri* 55730, generating the daughter strain *L. reuteri* DSM 17938, nearly identical in term of physiological attributes [8]. Plasmids are absents in the 6475 genome. *L. reuteri* genomes have a similar average chromosomal GC content (38%) consistent with previously described *L. reuteri* strains and other lactobacilli [21], [27]. The GC content of the cured plasmids was slightly higher, ranging from 39% for pLR581 to 41% for pLR585.

Both strains possess a core genome containing approximately 1600 genes, and representing approximately 70% of the total genetic content. However, as many as 289 genes (including pseudogenes) are present in the genome of strain 6475 and absent in the genome of strain 55730, while up to 700 genes (including pseudogenes) are present in the 55730 genome and absent in 6475. Comparisons of the percentages of the different clusters of orthologous groups (COG) categories revealed that the genome of strain 55730 was enriched with respect to genes involved in cell wall/membrane/envelope biogenesis, transcription, cell motility, general function, but also in genes of unknown function when compared to 6475 (**Figure S1**). The genome of strain 6475 is slightly enriched in DNA replication/recombination/repair COG categories. The genome of strain 55730 is characterized by the presence of more than 400 genes annotated as transposases, indicating extensive genomic rearrangements (**Figure S2**) in this strain. Several gene truncations are present in the 55730 genome mainly due to the presence of insertional elements.

Deviation from the average GC content (>10% from the average whole genome GC% content of 39%) indicates that genes in both *L. reuteri* genomes may have been acquired by horizontal gene transfer [21]. A genomic island encoding the propanediol utilization and vitamin B_12_ operons with a very low GC content (GC content close to 25%), is present in both strains 6475 and 55730, as described previously in other *L. reuteri* strains [21]. A glycerol kinase gene (GC content of 51%) is also present in both strains. A neopullulanase in strain 6475, and a dextranase, L- ribulose-5-phosphate-4 epimerase (GC content of 53%), several glycosylhydrolases and hypothetical proteins (GC content between 26–29%) predicted to encode the synthesis of exopolysaccharide (EPS) in strain 55730 have been also probably acquired from other microorganisms such as Proteobacteria and other lactic acid bacteria.

### Metabolic Modeling of *L. reuteri*

Based on whole genome comparisons, metabolic models representative of each group of human-derived strains (6475 and 55730) were also built. The metabolic model of 6475 includes 563 genes, similar to the metabolic model of *L. reuteri* JCM 1112. The metabolic model of 55730 includes a total of 623 genes. Compared to the metabolic model of strain 6475, 109 genes unique to 55730 have been added to the 55730 metabolic model. Compared to the metabolic model of strain 6475, 51 genes not present in the 55730 genome have been excluded. Overall, this model differs in the pathways related to carbohydrate metabolism (described in more detail in the paragraph “Carbohydrate Metabolism”), with the addition of several genes involved in the tricarboxylic acid (TCA) cycle, polysaccharide transport and exopolysaccharide synthesis in strain 55730. Strain 6475 was predicted to be able to synthesize 10 amino acids (arginine, alanine, asparagine, aspartate, cysteine, glutamine, glycine, proline, serine, and lysine). Additional enzymes involved in amino acid synthesis (threonine synthase for instance) were present in strain 55730 (described in paragraph “Probiotics as Nutrient Factories”). Both strains were predicted to synthesize several vitamins *de novo* including vitamin B_12_ and folate, but only strain 55730 has a complete pathway for thiamine biosynthesis (described in more details in paragraph “Probiotics as Nutrient Factories”). A comprehensive pan-metabolic map in which common pathways are differentiated from strain-specific pathways is provided in **Figure S3**.

### Transcriptome Comparisons: Gene Expression Profiling of both Probiotic Strains

Transcriptomes of strains 55730 and 6475 were monitored at different time points in order to get an overview of various pathways active during specific growth phases. Genes expressed and detected in microarrays were then mapped to the pan-metabolic map of the two strains for easier visualization and comparison (transcriptome and genome-based mapping), and are provided in **Figures S4 and S5**. Overall, a higher number of genes (n = 643) were differentially expressed in strain 6475 between the early log phase at 8 h and the late stationary phase, compared to strain 55730 in which 322 genes were differentially expressed between these two time points (fold-change >1.5, P value<0.05). Several metabolic pathways were more highly expressed in the same growth phase for both strains. For example, genes involved in fatty acid, peptidoglycan, and ribosomal synthesis, as well as carbohydrate metabolism were generally highly expressed during the early log phase (T8). In the stationary phase (16 h and 24 h), highly up-regulated genes are linked to stress-related functions or hypothetical proteins. The largest differences in transcription between the two strains were observed in pathways related to carbohydrate metabolism, vitamin B_12_ synthesis, and conversion of 3-hydroxypropionaldehyde (reuterin) to 1,3 propanediol in the late stationary phase. In strain 6475, numerous hypothetical proteins were highly expressed in this phase. Other interesting differences between the transcriptomes of the two *L. reuteri* strains include the arginine metabolic pathway. Arginine degradation via the arginine deaminase (ADI) pathway was highly expressed in the stationary phase for strain 6475, while in contrast, arginine biosynthesis was strongly up-regulated in strain 55730. In strain 55730, metabolic pathways strongly up-regulated in the stationary phase included the purine and glutathione synthesis pathways.

### Carbohydrate Metabolism - Basic Metabolism affecting Survival and Persistence

The capacity of a probiotic strain to adapt to nutrient availability and environmental conditions in various microhabitats of the GI tract is of utmost importance for their residence time and survival. *L. reuteri* is a heterofermentative species, which was confirmed in strain 55730 by the presence of both the phosphoketolase and Embden-Meyerhof pathways [28]. Both strains possess several genes that could be deployed to utilize human milk oligosaccharides (HMOs) (fucose transporter, beta-galactosidase, glucose-6 phosphate deiminase, deacetylase). Both genomes encode genes responsible for the degradation of 1,2 propanediol, a major by-product of the anaerobic degradation of rhamnose and fucose, which are common sugars in HMOs and plant cell walls, bacterial exopolysaccharides, and glycoconjugates of intestinal epithelial cells [29].

**Figure 1:**
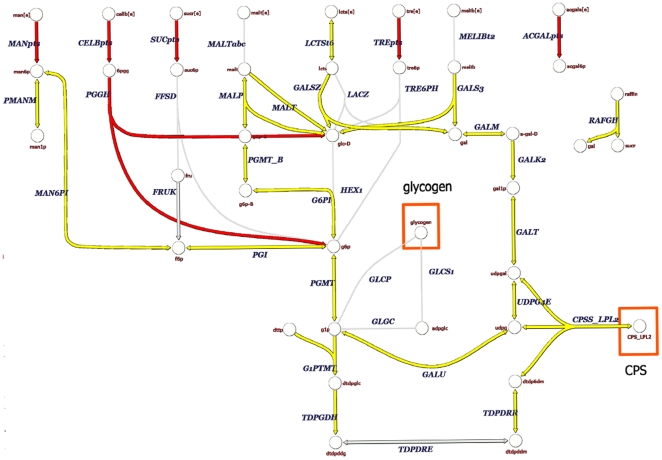
(Poly)saccharide metabolism in *L. reuteri* ATCC 55730 and ATCC PTA 6475. Metabolic reactions present in both strains are highlighted in dark grey. Metabolic reactions only present in *L. reuteri* ATCC 55730 are highlighted in black. Abbreviations are indicated for reactions present at least in one strain. [e]: extracellular; 6pgg: 6-phospho-beta-D-glucosyl-(1,4)-D-glucose; acgal6p: N-acetylgalactosamine 6-phosphate; acgala: N-Acetyl-D-galactosamine; ACGALpts: N-acetylgalactosamine PTS; a-gal-D: alpha-D galactose; CELBpts: cellobiose transport via PEP:Pyr PTS; cellb: cellobiose; CPS_LPL2: capsular polysaccharide linkage unit, LPL specific; CPSS_LPL2: CPS synthase complex, LPL specific; dtdp6dm: dTDP-6-deoxy-L-mannose; dtdpddg: dTDP-4-dehydro-6-deoxy-D-glucose; dtdpddm: dTDP-4-dehydro-6-deoxy-L-mannose; dtdpglc: dTDPglucose; dttp: dTTP; f6p: D-Fructose 6-phosphate; fru: D-Fructose; FRUK: fructokinase; g1p: D-Glucose 1-phosphate; G1PTMT: glucose-1-phosphate thymidylyltransferase; g6p: D-glucose 6-phosphate; g6p-B: beta-D-glucose 6-phosphate; G6P: Glucose-6-phosphate isomerase; gal: D-galactose; gal1p: alpha-D-Galactose 1-phosphate: GALK2: galactokinase; GALM: aldose 1-epimerase; GALS3: a-galactosidase (melibiose); GALSZ: beta-galactosidase; GALT: galactose-1-phosphate uridylyltransferase; GALU: UTP-glucose-1-phosphate uridylyltransferase; glc-D: D-glucose; lcts: lactose; LCTSt6: lactose transport in/out via proton symport; MALP: maltose phosphorylase; malt: maltose; MALT: alpha-glucosidase; man: D-mannose; man1p: D-mannose 1-phosphate; man6p: D-mannose 6-phosphate; MAN6PI: mannose-6-phosphate isomerase; MANpts: D-mannose transport via PEP:Pyr PTS; melib: melibiose; PGGH: 6-phospho-beta-glucosidase; PGI: glucose-6-phosphate isomerase; PGMT: phosphoglucomutase; PGMT_B: b-phosphoglucomutase; PMANM: phosphomannomutase; raffin: raffinose; RAFGH; raffinose galactohydrolase; suc6p: sucrose 6-phosphate: SUCpts: sucrose transport via PEP:Pyr PTS; sucr: sucrose; TDPDRE: dTDP-4-dehydrorhamnose 3,5-epimerase; TDPDRR: dTDP-4-dehydrorhamnose reductase; TDPGDH: dTDPglucose 4,6-dehydratase; tre: trehalose; tre6p: alpha'-trehalose 6-phosphate; TREpts: trehalose transport via PEP:Pyr PTS; udpg; UDPglucose; UDPG4E: UDPglucose 4-epimerase; udpgal: UDPgalactose. The red boxes depict compound that can be incorporated in biomass.

**Figure 2:**
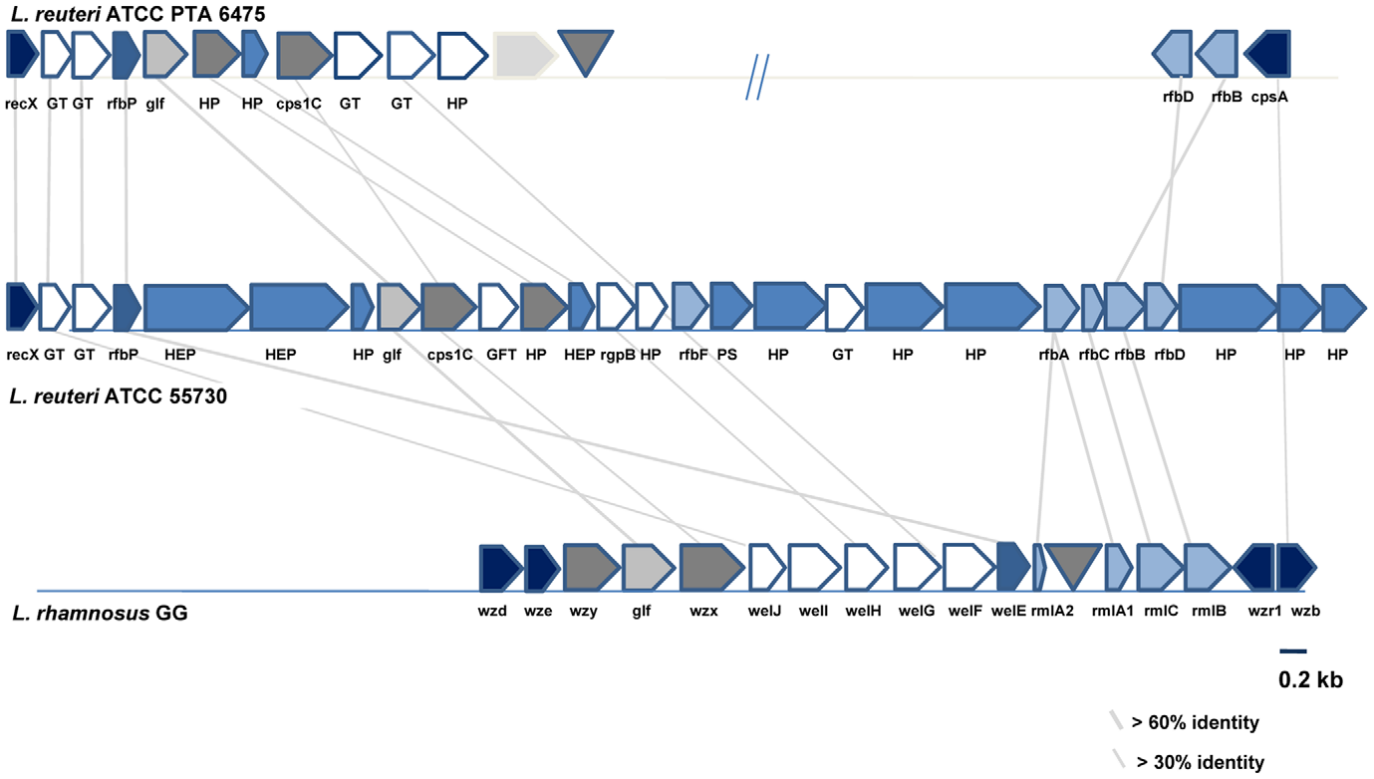
Exopolysaccharide gene cluster comparison in *L. reuteri* strain 6475, 55730, and *L. rhamnosus* GG. Genes encoding similar functions in EPS biosynthesis have a similar color. Genes indicated in dark blue encode proteins putatively involved in the regulation of EPS production and polymerization. Genes indicated in white encode putative glycosyltransferases. Genes indicated in light blue encode proteins involved in the biosynthesis of the dTDP-rhamnose precursor. Genes indicated in plain blue encode hypothetical proteins. Genes indicated in dark grey encode the putative polysaccharide transporter and polymerase. Transposases are indicated by a triangle. Intergenic regions are not represented at this scale. *cpsA*: cell envelope related transcriptional regulator; *cps1C*: polysaccharide biosynthesis protein; *GFT*: galactofuranosyltransferase; *GT*: glycosyltransferase; *HEP*: hypothetical extracellular protein; *HP*: hypothetical protein; *P*: permease; *PS*: polysaccharide synthesis protein; *recX*: regulatory protein; *rfbA*: glucose-1-phosphate thymidylyltransferase; *rfbB*: dTDP-glucose 4,6-dehydratase; *rfbC*: dTDP-4-dehydrorhamnose 3,5-epimerase; *rfbD*: dTDP-4-dehydrorhamnose reductase; *rfbF*: dTDP-rhamnosyl transferase; *rfbP*: undecaprenyl-phosphate galactose phosphotransferase; *rgbP*: group 2 glycosyl transferase; *r*mL*A1*, *r*mL*A2*: glucose-1-phosphate thymidyl transferase; *r*mL*B*: dTDP glucose 4,6-dehydratase; *r*mL*D*: dTDP-4-dehydrorhamnose reductase; *welE*: priming glycosyltransferase; *welF*, *welG*: glycosyltransferase; *welH*: α-L-rhamnose α-1,3-L-rhamnosyltransferase; *welI*, *welJ*: glycosyltransferase; *wzx*: flippase; *wzx*; non-specific protein-tyrosine kinase; *wzx*: polysaccharide transporter; *wzy*: polymerase.

Metabolic model comparisons of the two *L. reuteri* strains highlight the presence of additional pathways for carbohydrate uptake, degradation, and polysaccharide synthesis in strain 55730 (**Figure 1**). The presence of 3 subunits of the citrate lyase complex and a malate dehydrogenase suggests that strain 55730, but not 6475, had a partial TCA cycle, and is able to use citrate as an energy source (**Figure S6**). A lactoylglutathione lyase gene that can convert methylglyoxal into lactate is present only in strain 55730. Other genes present solely in 55730 related to carbohydrate metabolism include several glycosyltransferases, glycohydrolases and specific carbohydrate transporters for sucrose, trehalose and mannose. Interestingly, the glycohydrolases in strain 55730 formed a cluster of more than 25 genes that may contribute to EPS synthesis and is similarly organized compared to EPS-related genes described in other *Lactobacillus* strains, including *L. rhamnosus* GG. Although the start of this operon is similar in strain 6475, the presence of an insertional element in strain 6475 after the first 12 genes indicates that genomic rearrangements and gene loss have occurred. A comparison between the genetic organization of this EPS cluster in strain 55730, 6475 and *L. rhamnosus* GG is provided in **Figure 2**. Genes related to carbohydrate metabolism in strain 6475, but absent in strain 55730, included a neopullulanase (3 subunits) important in starch degradation, an arabinogalactan endo-1,4-beta-galactosidase, and an arabinose efflux transporter.

Differences highlighted in the genome sequences of *L. reuteri* strains 55730 and 6475 regarding carbohydrate metabolism were validated experimentally using a range of simple sugars and prebiotics as sole carbon sources by replacing glucose in the same medium. Strain 55730 demonstrated enhanced versatility with respect to proliferation with different carbohydrates as primary substrates, such as sucrose, raffinose, maltose, lactose, galactose, and galacto-oligosaccharides compared to strain 6475 (**Figure S7**). The last three sugars are major components of human breast milk, an environment from where both *L. reuteri* strains were isolated. Neither 55730 nor 6475 were able to ferment prebiotic fructans, and only strain 55730 was able to metabolize the prebiotic lactulose.

Differences in carbohydrate metabolism between the two strains were also visible based on gene expression patterns in different growth phases. For instance, a glucose uptake protein and a glucose hydrolase were highly expressed in 6475 during the early log phase. In contrast, ribose, ribokinase, and a deoxyribose transporter were strongly expressed in the early log phase in strain 55730. This result suggests that other carbon sources such as deoxyribose or ribose derivatives may have been used as alternative substrates by strain 55730. Confirming previous observations [28], both the phosphoketolase and Embden-Meyerhof pathways appear to be active during the logarithmic and stationary phases of both strains 55730 and 6475. Genes coding for EPS in 55730 were expressed throughout the different growth phases.

### Stress Resistance Mechanisms - Survival in the Midst of Physiologic Stressors

Various physiologic stresses affect the survival and functionality of microbes in the GI tract, and resistance to physiologic stresses is a desirable feature of probiotics. As examples, probiotics must face the gastric acidity, the antimicrobial activity of intestinal bile salts, and oxidative stress. Whole genome analysis demonstrated that human-derived *L. reuteri* strains are well equipped to respond to different sources of physiologic stress. Both strains possess a choloylglycine hydrolase, an enzyme that catalyzes the hydrolysis of conjugated bile salts in the intestine; however this gene is truncated in strain 55730. Growth in presence of bile has been shown to be effectively impaired in the 55730 strain only (S. Harpavat, personal communication). Both strains possess an arginine deiminase that can potentially produce ammonia (an alkaline compound) and ATP by converting arginine to ornithine [30]. Moreover, both strains have an arsenal of several genes that can counteract reactive oxygen species, including glutathione, methionine sulfoxide reductase, peroxiredoxin, peroxidase, thiol peroxidase, and glutathione disulfide reductase. Arginine degradation via the arginine deiminase pathway was highly expressed in both strains. Interestingly, this pathway was more highly expressed during the stationary phase in 6475, while it was more highly expressed during the early log phase in 55730.

### Adhesion Mechanisms - the Bacterial:Host Interface

The adhesion of probiotics to the intestinal mucosal surface could be a critical prerequisite for exerting beneficial effects to cognate mammalian hosts [31]. Mucosal adherence is considered one of the main selection criteria for potential probiotics, as adherence prolongs the persistence of microbes in the intestine. Both strains 55730 and 6475 possess a gene encoding a protein with very strong identity (>99%) to the 26 kDa mucus adhesion promoting protein (MapA) [32] or collagen binding protein CnBP [33]. A fibronectin binding protein A encoding gene exists and is nearly identical (99% identity) in both strains. These sequences do not contain any signal peptide. Several surface proteins with LPXTG sortase motifs were identified in both strains: three homolog encoding proteins with LPXTG sortase motifs were identified in both strains (two proteins of unknown function, and a 5′-nucleotidase). Additionally, two proteins of unknown function with LPXTG sortase motifs were identified solely in strain 55730, while four proteins with LPXTG sortase motifs were unique to strain 6475 (three proteins of unknown function, and a putative 6-aminohexanoate-cyclic-dimer hydrolase that contains a gamma-glutamyltransferase domain belonging to the AIG2-like family) (data not shown). A single gene encoding sortase A (*srtA*) was identified in both the 55730 and 6475 genome (>98% identity to each other).

### Probiotics as Nutrient Factories - Nutrition via the Microbiome

**Figure 3:**
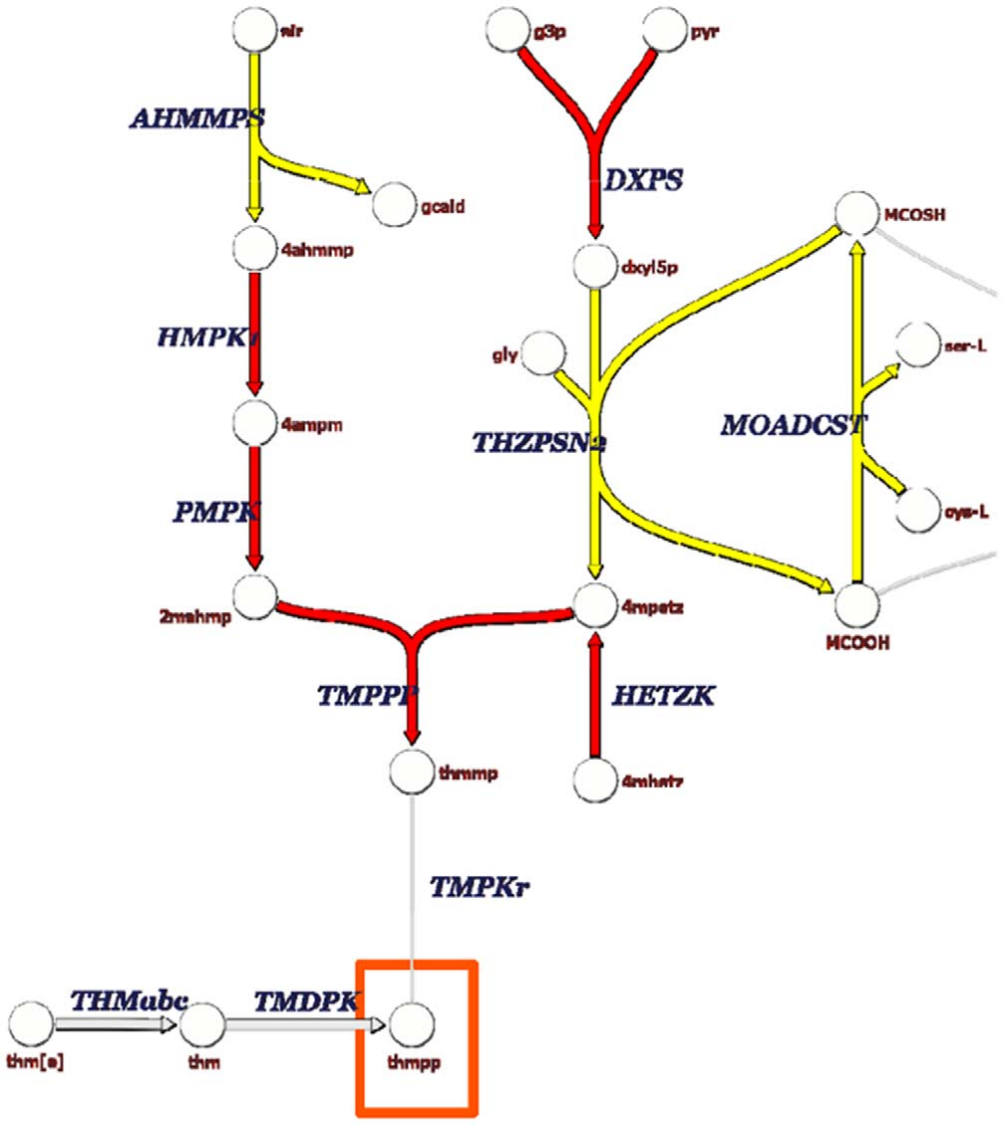
Thiamine biosynthesis pathway in *L. reuteri* ATCC 55730 and ATCC PTA 6475. The thiamine synthesis pathway is predicted to be complete in strain 55730 only. Metabolic reactions present in both strains 55730 and 6475 are highlighted in dark grey. Metabolic reactions present only in *L. reuteri* ATCC 55730 are highlighted in black. 2mahmp: 2-methyl-4-amino-5-hydroxymethylpyrimidine diphosphate; 4ahmmp: 4-amino-5-hydroxymethyl-2-methylpyrimidine; 4ampm: 4-amino-2-methyl-5-phosphomethylpyrimidine; 4mhetz: 4-methyl-5-(2-hydroxyethyl)-thiazole; 4mpetz: 4-methyl-5-(2-phosphoethyl)-thiazole; AHMMPS: 4-amino-5-hydroxymethyl-2-methylpyrimidine synthetase; air: 5-amino-1-(5-phospho-D-ribosyl)imidazole; DXPS: 1-deoxy-D-xylulose 5-phosphate synthase; dxyl5p: 1-deoxy-D-xylulose 5-phosphate; g3p: glyceraldehyde 3-phosphate; gcald: glycolaldehyde; gly: glycine; HETZK: hydroxyethylthiazole kinase; HMPK1: hydroxymethylpyrimidine kinase (ATP); MCOOH: MPT synthase small subunit MoaD; MCOSH: MPT synthase sulfurylated small subunit (MoaD-SH); MOADCST: MoaD:cysteine sulfur transferase; PMPK: phosphomethylpyrimidine kinase; pyr; pyruvate; thm: thiamine; thm[e]: thiamine (extracellular); THMabc: thiamine transport via ABC system; thmmp: thiamine monophosphate; thmpp: thiamine diphosphate; THZPSN2: thiazole phosphate synthesis; TMDPK: thiamine diphosphokinase; TMPKr: thiamine-phosphate kinase; TMPPP: thiamine-phosphate diphosphorylase. The red boxes depict compound that can be incorporated in biomass.

The synthesis of nutrients in the gastrointestinal tract by probiotics may enhance human nutrition by complementing dietary components and providing essential compounds for the human host and the microbiome [34]. Genome analysis revealed that 55730 and 6475 may be especially apt to synthesize vitamins essential to humans. *In silico* genome analysis revealed that both 6475 and 55730 have complete pathways for folate and vitamin B_12_ biosynthesis. Complementary analysis using mass spectroscopy had confirmed the presence of several folate derivatives, including long-chain folylpolyglutamates, in both strains (D. Saulnier, personal communication). Furthermore, a complete pathway for thiamine (Vitamin B_1_) production is predicted in strain 55730 only (**Figure 3**), and it is the first report of thiamine biosynthesis in lactobacilli. Metabolic pathways of other vitamins (vitamin K, biotin, pantotenate, riboflavin, pyridoxine) that have been described in other bacteria are absent in the genomes of strains 55730 and 6475.

Synthesis of essential amino acids for the host by probiotics is an interesting avenue that deserves further exploration. Eight amino acids are generally regarded as essential for humans: phenylalanine, valine, threonine, tryptophan, isoleucine, methionine, leucine, and lysine [35]. Additionally, cysteine (or sulphur-containing amino acids), tyrosine (or aromatic amino acids), histidine and arginine are required by infants and growing children [36], [37]. Strain 6475 is predicted to be able to synthesize 10 amino acids (arginine, alanine, asparagine, aspartate, cysteine, glutamine, glycine, proline, serine, and lysine), including three amino acids considered to be required by young children. In addition to the synthesis of these 10 amino acids, the presence of threonine synthase in strain 55730 suggests that the essential amino acid, threonine, can additionally be synthesized from the conversion of homoserine in this strain.

In addition to vitamins and essential amino acids, both *L. reuteri* strains produce lactate, an electron sink in the gut that can be further converted into butyrate, the main carbon source for the colonocytes, by other species of the microbiome [38]. Similar amounts (30–40 mM) of lactic acid were produced by strains 6475 and 55730 after 24 h of growth in a semi-defined medium (data not shown). However, qualitative differences in lactic acid isomers were found between the two strains. D- and L-lactic acid isomers were produced in equimolar amounts by strain 6475, while more L-lactic acid (ratio 3∶1) was produced by strain 55730 (data not shown). This difference may be attributed to the presence of a second L-lactate dehydrogenase gene in the 55730 genome. *L. reuteri* strains can produce the short chain fatty acid (SCFA), acetate, a substrate that is absorbed in the GI tract and enters the circulation to be metabolized by peripheral tissues [39].

### Production of Antimicrobial Compounds - Survival in the Microbial Community

**Figure 4:**
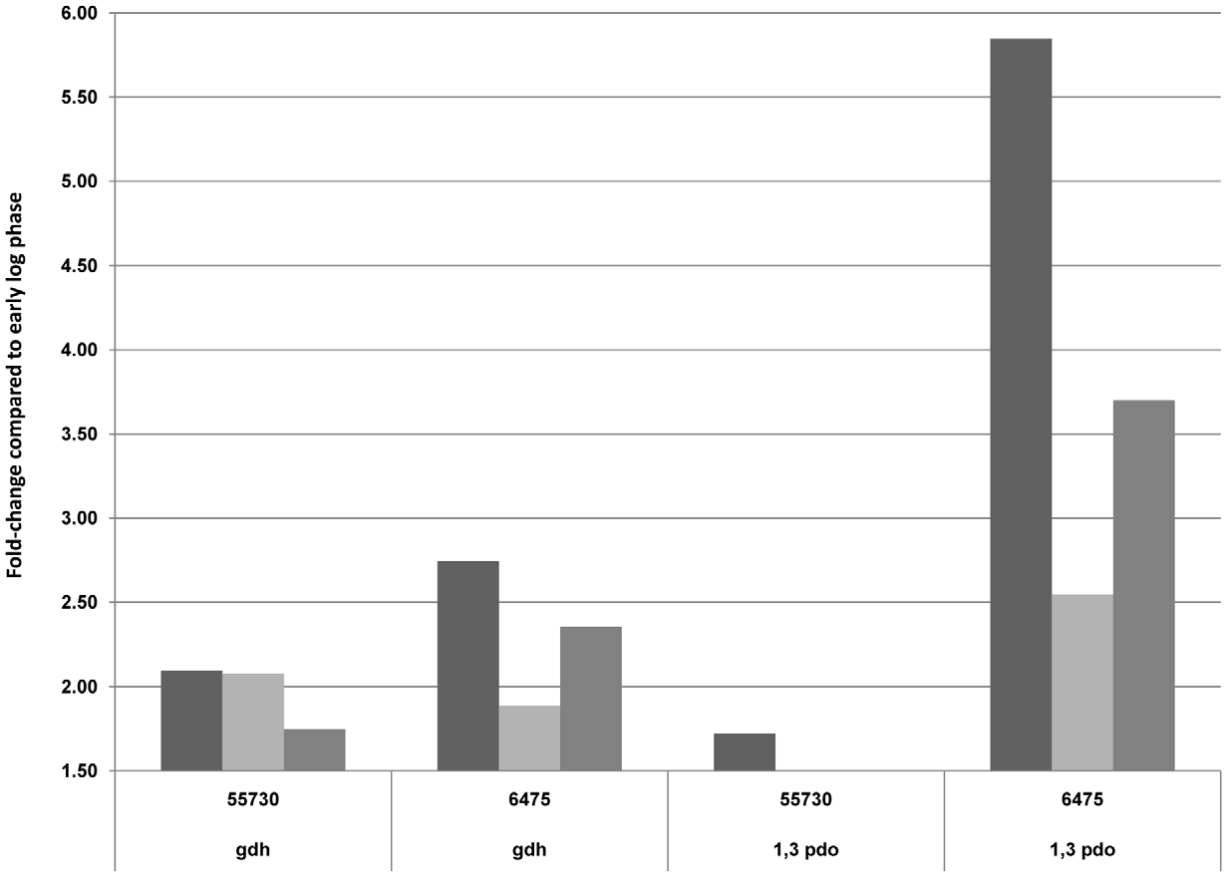
Transcription comparisons of genes involved in reuterin production and conversion in *L. reuteri* strains overtime. 12 h versus 8 h (dark grey), 16 h versus 8 h (light grey), and 24 h versus 8 h (medium grey). Strains were grown in a semi-defined medium in anoxic conditions at 37°C.

Like other probiotics and commensal microbes, *L. reuteri* produces antimicrobial factors, including lactate and reuterin. Strain 55730 produced up to three times more reuterin than strain 6475, but the reason for this difference is unknown [18]. The genes responsible for reuterin production such as the glycerol dehydratase (*gdh*) are part of the propanediol utilization (*pdu*) operon. The gene (*1,3 pdo*) encoding the enzyme responsible for the conversion of reuterin into 1,3 propanediol is 1,3 propanediol dehydratase, and this gene is positioned at another location in the genome. Genome comparisons between the different strains showed a similar gene organization of the *pdu* operon. All genes within this operon were 95–100% identical in strains 55730 and 6475, with the exception of the transcriptional regulator, *pocR* (74% identity between each strain), that has been shown to regulate reuterin production in these strains (Spinler et al, personal communication). In terms of gene transcription, the *pdu* operon was highly expressed throughout the logarithmic and stationary growth phases in both strains. Although *gdh* was not differentially expressed between the two strains, *1,3 pdo* was strongly up-regulated after 12, 16, and 24 h in strain 6475 only (**Figure 4**). The upregulation of *1,3 pdo* in strain 6475 probably accounts for the lack of stationary phase enhancement of reuterin production in this strain.

Besides lactic acid and reuterin, a new antimicrobial compound derived from the degradation of the protein MapA was recently reported in a porcine isolate of *L. reuteri* [40]. The purified 48 amino acid peptide of 5.3 kDa with antimicrobial activity was heat stable and possessed a strong pI (9.3), physical properties characteristics of bacteriocins. The processing or degradation of this peptide is not known. The presence of the same genomic sequence (100% identity) with the protein described by Bohle *et al* in both strains 6475 and 55730 suggests that this new antimicrobial agent could also be produced by these two strains.

### Mechanisms of Immunomodulation

Strains 55730 and 6475 are known to exert opposite immunomodulatory effects *in vitro* (see Lin et al [17]). Genome-wide analysis revealed that the genome of the immunostimulatory strain 55730 encodes the synthesis of putative compounds (EPS, cell surface protein containing five repetitive Rib motifs) that can be pro-inflammatory. Organization of the EPS is similar to an EPS described in the probiotic *L. rhamnosus* GG, and this gene complex has been associated with pro-inflammatory functions [41]. However, a galactose-rich EPS are synthesized by *L. rhamnosus* GG, but genomic analysis of the operon suggests that a rhamnose-rich EPS may be synthesized by strain 55730. Besides this EPS, a gene encoding a cell surface protein containing five repetitive Rib motifs is only present in strain 55730. This gene has a strong similarity to a surface protein in *Streptococcus pyogenes* thought to confer protective immunity [42], [43].

*In silico* analysis of anti-inflammatory strain 6475 did not reveal the presence of genes previously known to play a role in suppression of immune responses. Previous experiments conducted with the supernatant of 6475 grown in the same semi-defined medium and harvested in late stationary phase has demonstrated that this strain strongly reduced TNF production by lipopolysaccharide (LPS) activated monocytoid cells [17]. In an effort to focus on genes more highly expressed during this specific growth phase, we compared gene expression profiles of strain 6475 in different growth phases using the same medium and environmental conditions. We also excluded genes that were strongly up-regulated in the stationary phase using the immunostimulatory strain 55730. The most highly expressed genes unique to strain 6475 encode proteins involved in DNA repair, competence, folate biosynthesis, or proteins of unknown function (**Table 1**). Phenotypic analyses of strains containing a genetic insertion in some of these genes have validated their predicted role in immunomodulation (D. Saulnier, personal communication).

**Table 1:** Genes significantly up-regulated in the stationary phase (24 h) compared to early log phase (8 h) for *L. reuteri* ATCC PTA 6475.

Microarray Probe ID	Protein ID	Name	Fold-change 24 h/8 h*
**Protein with unknown function**			
NT01LR0789	GI:227531231	Hypothetical protein	43.3
NT01LR0977	GI:227532502	Hypothetical protein	32.9
NT01LR1311	GI:227532590	Hypothetical protein	29.0
NT01LR0293	GI:227532398	Hypothetical protein	19.3
NT01LR1962	GI:227532487	Hypothetical protein	15.0
NT01LR0916	GI:227531908	Hypothetical protein	11.7
**Competence**			
NT01LR0625	GI:227530733	ATP-dependent ClpP protease proteolytic subunit	24.7
NT01LR1937	GI:227530859	Competence protein CoiA-like family	15.3
NT01LR1936	GI:227530860	Possible dithiol-disulfide isomerase	13.6
**Vitamin and cofactor metabolism**			
NT01LR1511	GI:227532295	Putative tetrahydrofolate synthase	20.6
NT01LR1371	GI:227532039	Cobalamin biosynthesis protein CbiD	14.5
NT01LR1375	GI:227532035	Possible threonine-phosphate decarboxylase (EC:4.1.1.81)	12.0
NT01LR1372	GI:227532038	Cobalt-precorrin-8X methylmutase	11.6
**DNA repair**			
NT01LR1510	GI:227532296	DNA repair protein *radC*	18.1
**Carbohydrate metabolism**			
NT01LR1312	GI:227532591	Ribose operon repressor	17.3
**Prophage**			
NT01LR1675	GI:227531144	Hypothetical protein	15.7
NT01LR1673	GI:227531141	Conserved hypothetical protein	15.6
NT01LR1676	GI:227531146	Prophage Lp1 protein 18	15.1
NT01LR0827	GI:227531270	Hypothetical extracellular protein	12.7
**Thymidine metabolism**			
NT01LR1345	GI:227532065	thymidine kinase	15.6

*Only genes significantly up-regulated more than 11.5-fold are represented. The genes indicated were not present or not differentially expressed at this phase of growth in *L. reuteri* strain ATCC 55730. The strains were grown in a semi-defined medium at 37°C in anoxic conditions.

## Discussion

Bacterial species that are considered commensal microbes may contain a number of beneficial features that enhance host physiology. Probiotics deserve attention as beneficial microbes with specific features that promote human health and ameliorate or prevent disease. However, the functional diversity within individual bacterial species considered to be probiotics has only recently been appreciated. In this report, we describe significant differences in the genome structure and whole genome gene expression profiles of one probiotic species *Lactobacillus reuteri*. Potentially host-beneficial or probiotic features were explored in detail using the bio-informatic methods of metabolic modeling. With respect to carbohydrate substrate utilization and metabolism, one strain, 55730, appears to be significantly more versatile with practical consequences in terms of host survival and persistence. Both strains explored in this study appear to have evolved multiple mechanisms of physiologic stress resistance in the gut. Examination of the nutrient production phenotype has yielded insights into the abilities of both strains to produce vitamins and essential amino acids. These strains have different phenotypes with respect to antimicrobial agent production, affecting microbe∶microbe interactions in the gastrointestinal tract. Finally, a stark contrast with respect to features of immumomodulation (TNF stimulation in the case of 55730 and TNF suppression in the case of 6475) may depend on multiple differences in genetic features.

Although the two *L. reuteri* strains belong to the same species, genome comparisons revealed that only 70% of the genes were shared between strains 55730 and 6475. The genomic and functional diversity within a single probiotic species emphasizes the need for strains to be clearly distinguished and verified in experimental studies. Meta-analyses of probiotics in clinical trials have often “lumped” species and strains together with the result that effects may be difficult to interpret [44]. The two metabolic models described in this report share many overlapping genes (more than 90% of the genes), but many genes unique to strains 55730 or 6475, lack known metabolic functions and may be entirely absent from current metabolic models. Most genes included solely in one of the strains were probably acquired by horizontal gene transfer due to differences in average % GC content between these genes and the harboring genomes. The presence of these genes may confer specific advantages in changing environments as documented in other bacteria [45]. Extensive evidence of horizontal gene transfer by bacteriophages or conjugation has been documented in *Lactobacillales* using genomic comparisons, and has been shown to be important for specific niche adaptation in probiotic bacteria [19]. Pathways rapidly acquired in prokaryotes are usually related to cofactor/vitamins, glycans, and environmental information processing/signaling pathways [46]. These observations seem to apply to *L. reuteri* strains. For example, *L. reuteri* 55730 is predicted to metabolize thiamine, synthesize several polysaccharides, and possesses additional genes in the TCA cycle compared to 6475. The presence of these genes in the TCA cycle in *L. reuteri* has been demonstrated indirectly previously [47]. In this study, Kaneuchi *et al* tested 39 strains and 59% of these strains were able to produce succinate from citrate, confirming that this feature is common in this species. Citrate is present in substantive amounts in the human breast milk and could provide a competitive advantage to this strain in this environment, more specifically at the time of delivery when citrate concentration increases [48].

*In silico* genome analysis revealed that both strains 55730 and 6475 possessed common factors attributed to survival in the GI tract. The role of genes involved in bile acid resistance have also been confirmed using gene expression and targeted mutagenesis in strain 55730 [49]. The presence of a complete choloylglycine hydrolase gene in strain 6475 suggests that this gene could be an additional asset to counteract the antimicrobial activity of bile salts, but that strain 55730 may be more susceptible to bile salts or may contain some other feature of bile salt resistance. Genome analysis suggested that these strains may have a competitive advantage in human breast milk by being able to utilize sugars commonly found in milk (lactose, galactose, galacto-oligosaccharides). Their genomes encode several genes that could participate in the (partial) degradation of complex HMOs, as demonstrated previously for bifidobacteria isolated from the same environment [20]. Although degradation of complex HMOs by *L. reuteri* strains has not been studied, this metabolic feature is biologically relevant as *L. reuteri* has been found in the breast milk of 15% of nursing mothers worldwide [3], [50]. Transcriptome analysis has demonstrated that the products of degradation of nucleic acid hydrolyis, such as ribose, are also found in breast milk and may be an important carbon source for certain *L. reuteri* strains. Products of degradation of nucleic acid metabolism may also be a major source of these sugars *in vivo*, generated from bacterial lysis or the shedding of enterocytes degraded by bacterial or host nuclease. The intake of ribose has been confirmed experimentally for the strain 55730 [8] and 6475 (S. Roos, unpublished results).

Adhesion to the mucosal surface of the GI tract may improve the beneficial effects of probiotics for the host. Both strains 55730 and 6475 possessed a gene encoding a protein with homology to a mucus adhesion-promoting protein in *L. reuteri* R104 [32]. Purified MapA protein was able to bind to Caco-2 human colonocytes, and this binding inhibited the adhesion of several *L. reuteri* strains (including *L. reuteri* DSM 20016, highly similar to strain 6475) in a concentration-dependent manner [51]. A gene encoding sortase A could facilitate cell adhesion by promoting cell surface expression of different proteins, and this gene was identified in both strains 55730 and 6475. This enzyme might be essential to anchor cell surface proteins that contain the sorting signal LPXTG motif, as shown previously for several microorganisms [52], [53]. In strain 55730, a complex EPS is predicted to be synthesized, and may improve adherence and gut colonization as elegantly demonstrated for other indigenous microbes such as *Bacteroides fragilis* [54]. Further characterization of these proteins may expand our understanding of how probiotic strains adhere to intestinal epithelial cells and mucins.

In humans, it has been estimated that the liberation of energy by the commensal microbiota is approximately 10% of the energy absorbed in the diet, but it is highly dependent upon the nature of the diet [55]. Both strains were predicted to produce essential nutrients for the host (amino acids, vitamins). Previous studies have demonstrated that other *L. reuteri* strains such as JCM 1112 are able to synthesize vitamin B_12_, a rare characteristic among lactic acid bacteria [56]. This synthesis was explained by the presence of a large genomic island containing more than 25 genes for cobalamin synthesis that was probably transferred from gram-negative bacteria [21], [57]. A similar organization of this vitamin operon also exists in strains 6475 and 55730, suggesting that both strains are able to produce this metabolite. *In silico* genome analysis revealed that both 6475 and 55730 have complete pathways for folate biosynthesis. Preliminary studies in our laboratory have shown that several folate derivatives can be effectively produced by *L. reuteri* 6475 and 55730. Interestingly, genome analysis in strain 55730 highlighted the presence of a complete pathway for thiamine biosynthesis. Further studies must confirm whether thiamine is effectively produced and secreted in this strain. Thiamine biosynthesis has been well studied in other prokaryotes, including *E. coli*, *Salmonella*, and *Bacillus subtilis* [58], and complete pathways have been described in bifidobacteria isolated from human breast milk [20]. However, thiamine biosynthesis has not been described in *Lactobacillus* species, but it does not seem that the thiamine synthesis pathway in strain 55730 has been acquired from other bacteria. The genes that are part of the thiamine synthesis pathway in 55730 are not located in a single operon, but in multiple locations across the genome. Some of the genes of this pathway seem to have been lost in the 6475 strain. Predicted pathways for the production of essential amino acids were also identified, which could enhance the beneficial effects of probiotic strains. Both strains 55730 and 6475 possessed a pathway for the *de novo* synthesis of the essential amino acid lysine. It has been estimated that between 1–20% of circulating plasma lysine, urinary lysine and body protein lysine is derived from intestinal microbial sources [59] Both strains possess a pathway for the *de novo* synthesis of arginine, another essential amino acid for human infants. Essential nutrients and vitamins that can be produced by probiotics may warrant further studies and consideration as dietary strategies for vitamin- or amino acid-deficient populations. Furthermore, synthesis of lactate and acetate by *L. reuteri* can contribute important energy sources in the gastrointestinal tract, the conversion of lactate or acetate by the gut microbiota can lead to the production of butyrate and its trophic effects such as increased intestinal epithelial cell proliferation [60].

Production of antimicrobial compounds such as reuterin may facilitate prevention of gastrointestinal infections. A recent study highlighted that most human-derived *L. reuteri* strains are able to produce reuterin, in contrast to most *L. reuteri* strains isolated from rodents [5]. Whether reuterin synthesis provides a competitive advantage to human-derived *L. reuteri* strains remains to be determined. When cultured in a rich medium, strain 55730 produces up to three times more reuterin than 6475, both in planktonic culture and biofilms [13], [18]. By transcriptome analysis, all genes of this operon were expressed, but no differences in the transcription of *pocR* and *gdh* were observed. These results suggest that the propanediol utilization operon is expressed when *L. reuteri* strains are grown in the absence of glycerol, although production of reuterin does not occur when this compound is absent [61]. These findings suggest that this pathway may have other roles in *L. reuteri*. Additionally, the gene encoding the enzyme thought to be responsible for reuterin conversion to 1,3 propanediol was strongly up-regulated after 12, 16, and 24 h in strain 6475, but this gene induction was not observed for 55730. Differences in 1,3 *pdo* expression might explain differences of reuterin production between the two *L. reuteri* strains when grown in rich media.

Mechanisms of immunomodulation by probiotics is an area of research that has received ample attention [62], and immunomodulatory features distinguished strains 55730 and 6475 [16], [17]. Strain 55730 strongly stimulates TNF-production in LPS-activated monocytoid cells, while strain 6475 suppresses TNF-production under the same conditions. Using whole genome comparisons with well-characterized bacterial immunomodulins, putative genes and operons responsible for differences in immunomodulation between strains 55730 and 6475 were identified. Genes encoding the putative synthesis of EPS with a comparable organization in other lactobacilli warrant further characterization. The EPS is predicted to be constituted of repetitive units constituted of rhamnose, galactofuranose, galactopyranose, and N-acetylglucosamine. EPS and capsular polysaccharides have been characterized in other probiotic lactobacilli and linked to the stimulation of the immune system [63], [64], [65]. EPS produced by *L. rhamnosus* RW-9595M stimulates TNF production by macrophage-like RAW 264.7 cells [66], and shares strong similarities of organization with EPS in *L. rhamnosus* GG and *L. reuteri* 55730. Genes encoding EPS were expressed in strain 55730 throughout different growth phases in a semi-defined medium. In contrast, no candidate genes related to the global immunosuppresion or TNF inhibition were identified in the genome, transcriptome or metabolic model of strain 6475. Characterization of these candidate genes and their possible roles in immunomodulation is currently ongoing.

Genomic analysis, transcriptomic comparisons, and metabolic model reconstruction of two different human-derived *L. reuteri* strains provide a comprehensive overview of the metabolic capacity of one probiotic species. This work has important implications for the fields of probiogenomics and metagenomics, and functional studies of reference genomes and the mammalian microbiome will require deeper insights into metabolic models based on patterns of gene expression. In light of the explosion of metagenomics data as a result of global collaboration in the IHMC, metabolic reconstruction of an indigenous microorganism such as *L. reuteri* will lead investigators to understand the multiple metabolic pathways and functional capacity of a single commensal species and potential interactions between beneficial microbes and the human host. The many differences highlighted by comparisons of two groups of *L. reuteri* strains isolated from distinct niches (breast milk, gastrointestinal tract and the oral cavity) generate more questions about the ubiquity and possible mechanisms of probiosis in different body sites. Expression of specific genes and activation of specific metabolic pathways might allow microbes to thrive and survive in different environments. Computational reconstruction and analysis of cellular models of microbial metabolism is one of the great success stories of systems biology, and much more needs to be learned from the many microbial components of mutualistic microbiomes that inhabit mammals.

## Materials and Methods

### Whole Genome Sequencing of *L. reuteri* Genomes

As part of the HMP, four human-derived *L. reuteri* strains (*L. reuteri* ATCC PTA 6475 = MM4-1A, *L. reuteri* ATCC PTA 4659 = MM2-3, *L. reuteri* ATCC 55730 = SD2112, and *L. reuteri* CF48-3A) were sequenced at the Human Genome Sequencing Center at Baylor College of Medicine as described previously Nelson et al [1]. The genomic DNA was prepared from a single bacterial isolate. The sequence generated included at least 10-fold coverage of 454-FLX (Roche, Lifesciences, Brandford, CT) fragment data, at least 8-fold clone coverage of 454-FLX paired-end data, and at least 10-fold coverage of Illumina (Illumina, San Diego, CA) data. The 454 sequence was assembled using the 454 Newbler assembler version 2.3. The contigs from the Newbler assembly were aligned to the Illumina/Solexa data with mapping tools such as Mosaic and Crossmatch and these data were used for error correction for this version of the draft assembly. This draft assembly meets the HMP draft quality standards (more than 90% of the genome is included in contigs, more than 90% of a core set of bacterial genes are found with >30% identity and >30% length; more than 90% of the bases in the assembly have more than 5-fold sequence coverage, the contig N50 length is greater than 5 kb, the scaffold N50 length is greater than 20 kb, and there is less than 1 gap per 5 kb. Annotation was added to the contigs in April 2009. All whole genome shotgun sequences were deposited in Genbank (*L. reuteri* ATCC 55730: Genbank accession number ACGW00000000.2, improved assembly high quality draft, genome coverage = 63×, 14 contigs; *L. reuteri* CF48-3A: Genbank accession number ACHG00000000.1.2, draft assembly, genome coverage = 60×, 244 contigs; *L. reuteri* ATCC PTA 6475: Genbank accession number ACGX00000000.1, draft assembly, genome coverage = 63×, contigs; *L. reuteri* ATCC PTA 4659: Genbank accession number ACLB00000000.1, draft assembly, genome coverage = 37×, 167 contigs).

Whole genome sequences of human-derived *L. reuteri* strains were compared using the Integral Microbial Genome (IMG) Platform (http://img.jgi.doe.gov/) [67]. IMG generated annotation consists of protein family and domain characterization based on Clusters of Orthologous Groups (COG) [68], Pfam [69], TIGRfam and TIGR role categories [70], and Interpro domains [71]. LPXTG motifs were searched in the complete genome of *L. reuteri* JCM1112 using the Augur software (http://bioinfo.mikrobio.med.uni-giessen.de/augur), and BlastP [72] was then run against the 6475 genome. LPXTG motifs have been previously described in strain 55730 [42]. A list of all the *L. reuteri* strains mentioned in this study is provided in table 2.

**Table 2:** List of bacterial strains that have been used in this study.

Name	Host	Origin	Isolation source	Clade according to Oh et al [6]
*L. reuteri* ATCC 55730	Human	South American	Breast milk	VI
*L. reuteri* CF48-3A	Human	Europe	Feces	VI
*L. reuteri* DSM 17938	Human	(daughter strains of *L. reuteri* ATCC 55730)		(VI)
*L. reuteri* ATCC 4659	Human	Europe	Breast milk	II
*L. reuteri* ATCC PTA 6475	Human	Europe	Breast milk	II
*L. reuteri* JCM 1112	Human	Europe	Feces	II

### Metabolic Model Construction of Two Different Probiotic Strains

The *in silico* reconstruction of the genome-scale metabolic networks of two human-derived *L. reuteri* strains was performed by implementing the AUTOGRAPH method [73]. This semi-automatic method combines orthology predictions with available curated metabolic networks to infer gene-reaction associations. Using this same methodology, a metabolic model was recently constructed for the type strain *L. reuteri* JCM1112 [56], based on the networks of *L. plantarum* [74], *Lactococcus lactis* [75], *Bacillus subtilis* [76], and *E. coli* [77]. Due to the obvious close proximity between all human-derived *L. reuteri* strains relative to members of different taxa, the manually curated metabolic network of JCM1112 was used as a template for the development of the genome-scale models for *L. reuteri* ATCC PTA 6475 and ATCC 55730. Pair-wise orthologous relationships between the query species and JCM1112 were established by comparing their genome sequences (retrieved in May 2009 from GenBank), resorting to the stand-alone version of Inparanoid (version 3.0) using BLOSUM80 as the substitution matrix [78]. The original gene-reaction association of the genes considered to be orthologous between the two strains was then transferred to the corresponding genes of the query species.

The fully automated version of the model was further curated by manual inspection of the list of gene-reaction associations, incorporating experimental evidence regarding carbohydrate utilization. With this purpose, the growth of *L. reuteri* 55730 and 6475 on different carbohydrates was measured for 24 h in LDMIII at 600 nm (OD_600 nm_) using commercially available sugars and well established prebiotics as previously described [79]. Simple carbohydrates tested consist of glucose, sucrose, lactose, raffinose, fructose, arabinose, maltose, mannose, arabinogalactan, starch and 1,2 propanediol (Sigma, St Louis, MO). Growth on following prebiotics as the sole carbon source were also tested: fructooligosaccharides (FOS, Beneo™ P95, Orafti, Belgium, 5% glucose, fructose and sucrose, degree of polymerization [DP] = 2–10), short-chain fructooligosaccharides (ScFOS, Actilight 950P, Beighin-Meiji, France, 5% glucose, fructose and sucrose, DP = 2–5), high-molecular weight inulin (Beneo™ HP, Orafti, 100% inulin, average DP = 23), galactooligosaccharides (Vivinal GOS, Friesland Food, partially dried by evaporation to form a syrup containing approximately 45% galactooligosaccharides, DP = 3–8, 15% lactose, 14% glucose, and 1% galactose).

The comparison of the newly obtained genome-scale metabolic models for *L. reuteri* ATCC PTA 6475 and ATCC 55730, along with the visualization of experimental data was carried out within the SimPheny™ software platform (Genomatica, Inc., San Diego, CA).

### Cell Harvesting and RNA Isolation for Transcriptome Comparisons of *L. reuteri* Strains

Culture stocks of *L. reuteri* 55730 or 6475 stored at −80°C were first cultured for 24 h on MRS agar at 37°C under anaerobic conditions (80% N_2_, 10% H_2_, and 10% CO_2_, MG-500, Microbiology International, Frederick, MD). A single colony was resuspended and cultured overnight in MRS broth (16–18 h). Bacteria were then resuspended to a concentration of ∼1.0×10^8^ cells/mL in 10 mL of a semi-defined medium LDMIII with 10 g/L of glucose as sole carbon source [13] and were incubated anaerobically up to 24 h. Samples were taken after 8 h (early exponential phase), 12 h (late exponential phase), 16 h (early stationary phase) and 24 h (late stationary phase). Optical density at 600 nm was measured using absorbance spectrometry to estimate growth phase. The experiments were performed in triplicate for each strain. A 10 mL cold fixative solution (100% [v/v] cold methanol) was added to quickly halt transcription. Samples were then centrifuged (10 min at 1,500× *g*), and the pellets were stored at −80°C prior to RNA isolation.

Frozen pellets were thawed on ice, and then resuspended in STE buffer (6.7% sucrose, 50 mM Tris [pH 8.0], 1 mM EDTA), harvested by centrifugation at 4000× *g* for 10 min, and resuspended in 100 µl of STE containing 5 µl of mutanolysin (5 U/µl, St Louis, MO). Cells were incubated at 37°C for 2 h. RNA was extracted using the RNeasy kit (Qiagen, Valencia, CA), and DNA was removed by adding RNase-free DNase (Qiagen, Valencia, CA). RNA concentrations were measured at 260 nm with the ND-1000 spectrophotometer (NanoDrop Technologies Inc., Wilmington, DE). The A_260_/A_280_ ratio was measured to determine the relative purity of the RNA. RNA samples were analyzed by 1% agarose gel electrophoresis to assess RNA quality.

For expression analyses, three biological replicates were performed with dye-swap experiments for each comparison. Following mRNA isolation, cDNA synthesis, labeling, and hybridization were performed as previously described [80].

### *L. reuteri* Strain-Specific Microarrays and Data Analysis/Visualization

Strain-specific two-color microarrays (“55730” or “6475” arrays) were used to compare the transcriptome of each strain at different phases of growth. The 55730 array has been described previously [49], and is based on the draft genome sequence of *L. reuteri* 55730 [42]. In brief, oligonucleotides (60-mers) were designed and synthesized for 1,864 open reading frames of this strain. The same technology was used for the 6475 array, and oligonucleotides (60-mers) were designed and synthesized for 1,966 open reading frames using OligoArray 1.0 software. Oligonucleotide design, synthesis, and array construction were performed at the Research Technology Support Facility at Michigan State University, East Lansing, MI. GenePix Pro 4.0.12 software was utilized for image analysis of the microarrays. We performed 18 arrays to study the transcriptome of each strain at different phases of growth. Information regarding the microarray platforms can be found at the NCBI Gene Expression Omnibus (GEO; http://www.ncbi.nlm.nih.gov/geo/) under GEO platform GPL6366 for the 55730 array or GPL7541 for the 6475 array. The complete set of microarray data for 55730 and 6475 can be found under the GEO series accession GSE24570 and GSE24415 respectively, or under the superseries GSE24572.

Data from custom microarrays for 55730 or 6475 were analyzed using R/Bioconductor (http://www.bioconductor.org/). The raw data in the .gpr files was normalized using statistical algorithms specifically designed for two color arrays implemented in Bioconductor packages of R. The raw signal intensity data was background corrected using the moving minimum algorithm. Print-tip loess is applied for Within Array normalization and quantile normalization is used for Between Arrays to normalize between chips. An expression value, in log2-scale, is obtained for each channel in the custom two color arrays. Each set (55730, 6475) was normalized and in the case of the comparison analysis, a combined expression set was generated. Null hypotheses were tested for each experiment. The null hypothesis states that there are no significant changes in gene expression between the treatment pairs. This comparison was done using Limma, which is also implemented in R/Bioconductor. It uses an empirical Bayesian method to estimate the variance in gene expression. Comparisons were made between the reference samples at times T8 and T12, T16, T24, respectively.

A comparison analysis was also run in the same manner as above, with the “between array normalization” that also excluded probe sets that were not common to both chips, thereby generating a combined expression set. The null hypotheses were tested using T8 as a common reference and contrasts were made at T12 or T16 or T24 between strain 55730 and strain 6475. The resulting list was filtered further using Microsoft Access to exclude any probe set with an identity score less than 30%. Scored lists were filtered again by adjusted p-value (<0.05) and fold change (1.5) and probe sets not meeting these criteria for both strains were excluded yielding a list of 910 probe sets.

The protein sequences of the two strains were linked with the probes (60 mer-sequences) of the strain-specific 55730 or 6475 microarray using the “many to many blasts” feature in DNAnnotator (http://bioinfo.bsd.uchicago.edu/blastall.htm).

Genes that were significantly up-or down-regulated were visualized on the genome of *L. reuteri* JCM112 using ERGO™ (Integrated Genomics Inc, Arlighton Heights, IL) for both 55730 and 6475 microarrays, using a cut-off of 80% identity and similarities with the probe sequences. These same genes were also visualized in the metabolic model using SimPheny (Genomatica, Inc., San Diego, CA).

## Supporting Information

Figure S1 **Comparison of genes representing different clusters of orthologous groups (COG) in** ***L. reuteri*** **ATCC 55730 and** ***L. reuteri*** **ATCC PTA 6475.** 1592 genes (69.2% of the genome) had a COG classification in *L. reuteri* ATCC 55730 (light grey), and 1495 genes (78.6% of the genome) in *L. reuteri* ATCC PTA 6475 (dark grey).
(TIF)

Figure S2 **Whole genome comparisons of** ***L. reuteri*** **ATCC 55730 and ATCC PTA 6475.** Comparisons were completed using the Artemis Comparison Tool (ACT) in IMG. For this analysis, the scaffolds of the 55730 and 6475 genome were combined and the chromosome replication initiation site was identified. Visual genome comparisons of the genomes of strains 55730 and JCM1112 were prepared by using ACT (BLASTN with a score cutoff of 1900, pair-wise genomic comparisons). Both sequences are read left to right from the predicted origin of replication. Homologous regions within the two genomes identified by reciprocal BLASTN are indicated by red (same orientation) and blue (reverse orientation) bars.
(TIF)

Figure S3 **Pan-metabolic model of** ***L. reuteri*** **ATCC 55730 and ATCC PTA 6475 based on whole genome sequences.** Metabolic reactions represented in yellow are present in both strains. Metabolic reactions represented in green are only present in *L. reuteri* ATCC PTA 6475. Metabolic reactions represented in red are only present in *L. reuteri* ATCC 55730. The vitamin B_12_ synthesis pathway is present in both strains but not represented on the map.
(TIF)

Figure S4 **Transcriptome comparison of** ***L. reuteri*** **ATCC 55730 in the early log and late stationary phase.** *L. reuteri* was grown in LDM at 37°C in anoxic conditions. Metabolic reactions in red represent genes significantly up-regulated (>1.5 fold, P<0.05) in the early log phase (8 h). Metabolic reactions in green represent genes significantly up-regulated (>1.5 fold, P<0.05) in the late stationary phase (24 h). N = 6. The vitamin B_12_ synthesis pathway is not represented on this map.
(TIF)

Figure S5 **Transcriptome comparison of** ***L. reuteri*** **ATCC PTA 6475 in the early log and late stationary phase.** *L. reuteri* was grown in LDM at 37°C in anoxic conditions. Metabolic reactions in red represent genes significantly up-regulated (>1.5 fold, P<0.05) in the early log phase (8 h). Metabolic reactions in green represent genes significantly up-regulated (>1.5 fold, P<0.05) in the late stationary phase (24 h). N = 6. The vitamin B_12_ synthesis pathway is not represented on this map.
(TIF)

Figure S6 **Prediction of additional enzymes tricarboxylic acid (TCA) pathway in** ***L. reuteri*** **ATCC 55730 and partial TCA pathway in** ***L. reuteri*** **ATCC PTA 6475.** Genes encoding the enzymes represented in blue are present in both *L. reuteri* ATCC 55730 and *L. reuteri* ATCC PTA 6475. Genes encoding the enzymes represented in orange are only present in *L. reuteri* ATCC 55730. EC:1.1.1.37: malate dehydrogenase; EC:1.2.4.1; pyruvate dehydrogenase (acetyl-transferring); EC:1.3.99.1: succinate dehydrogenase; EC:1.8.1.4: dihydrolipoyl dehydrogenase; EC:2.3.1.12: dihydrolipoyllysine-residue acetyltransferase; EC:4.1.3.6: citrate (pro-3S)-lyase (3 subunits represented by 3 different genes in *L. reuteri* 55730); EC:4.2.1.2: fumarate hydratase. Figure was obtained by projecting genes present in the TCA pathway in strain 55730 and 6475 via the Integral Microbial Genomes Platform: http://img.jgi.doe.gov.
(TIF)

Figure S7 **Final OD600 nm reached by** ***L. reuteri*** **ATCC 55730 (light grey) and** ***L. reuteri*** **ATCC PTA 6475 (dark grey) after 24 h of growth in LDM medium with 20 g/L of different carbon source.** Strains were grown in anoxic conditions at 37°C. FOS: fructooligosaccharide; ScFOS: fructooligosaccharides; Raftiline HP: long-chain inulin. Error bars represent standard deviations. Data represent the average of 3 biological replicates.
(TIF)
