# Supplementary figures and images for: Exploring Metabolic Pathway Reconstruction and Genome-Wide Expression Profiling in Lactobacillus reuteri to Define Functional Probiotic Features
Source: PLoS One. 2011 Apr 29;6(4):e18783. doi: 10.1371/journal.pone.0018783 (PMC3084715; doi:10.1371/journal.pone.0018783)

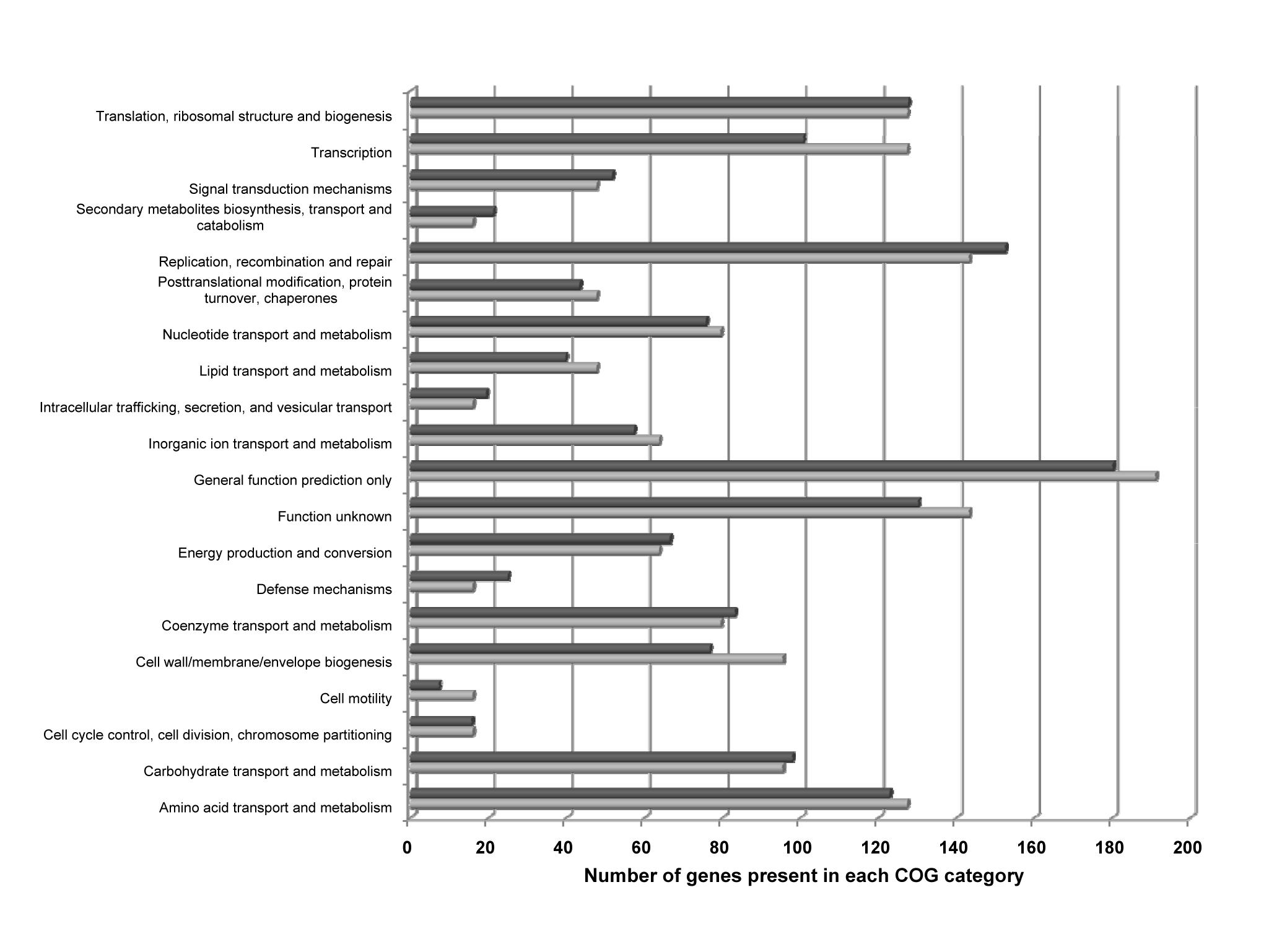

Supplement: Figure S1 — Comparison of genes representing different clusters of orthologous groups (COG) in L. reuteri ATCC 55730 and L. reuteri ATCC PTA 6475. 1592 genes (69.2% of the genome) had a COG classification in L. reuteri ATCC 55730 (light grey), and 1495 genes (78.6% of the genome) in L. reuteri ATCC PTA 6475 (dark grey). (TIF) [file pone.0018783.s001.tif]

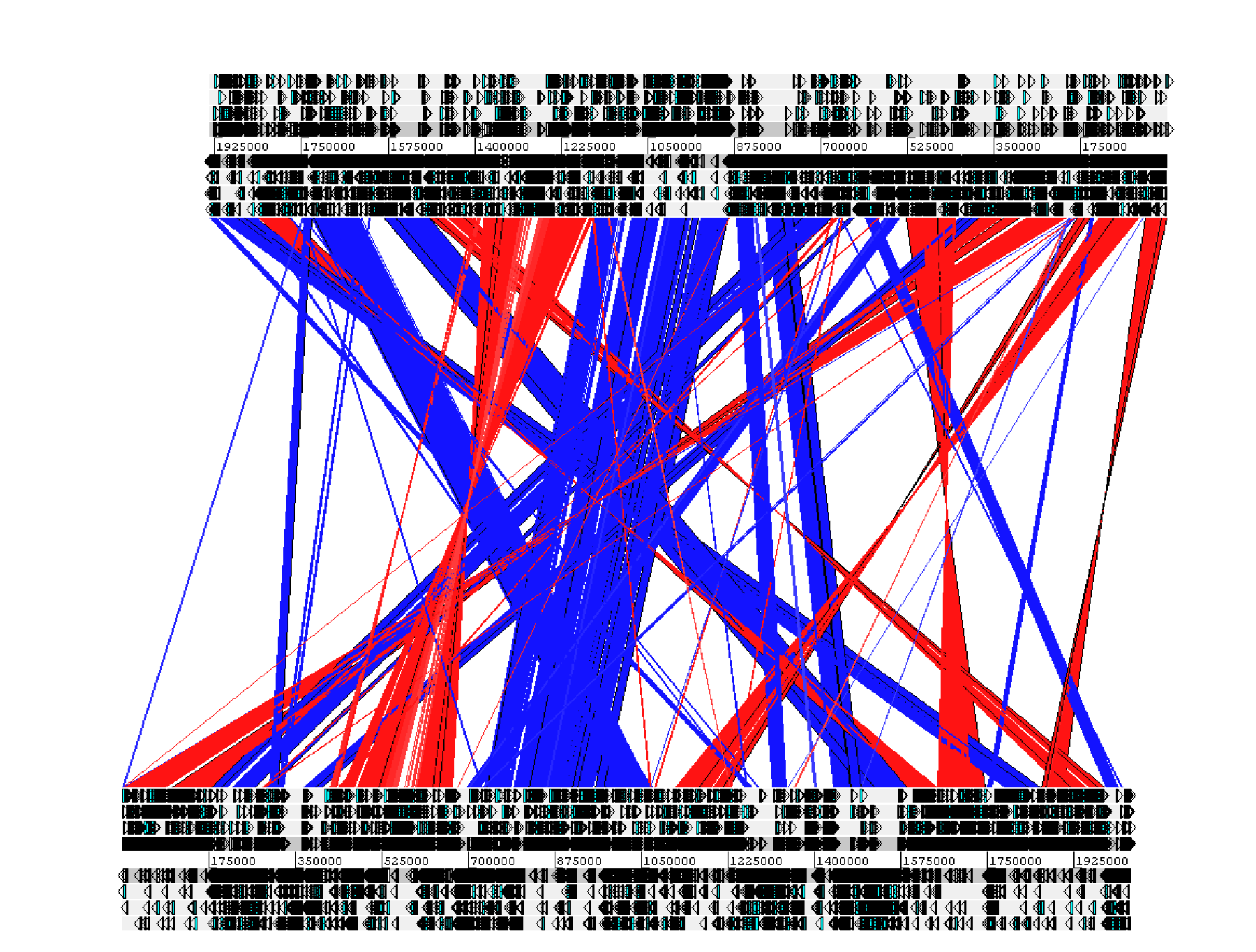

Supplement: Figure S2 — Whole genome comparisons of L. reuteri ATCC 55730 and ATCC PTA 6475. Comparisons were completed using the Artemis Comparison Tool (ACT) in IMG. For this analysis, the scaffolds of the 55730 and 6475 genome were combined and the chromosome replication initiation site was identified. Visual genome comparisons of the genomes of strains 55730 and JCM1112 were prepared by using ACT (BLASTN with a score cutoff of 1900, pair-wise genomic comparisons). Both sequences are read left to right from the predicted origin of replication. Homologous regions within the two genomes identified by reciprocal BLASTN are indicated by red (same orientation) and blue (reverse orientation) bars. (TIF) [file pone.0018783.s002.tif]

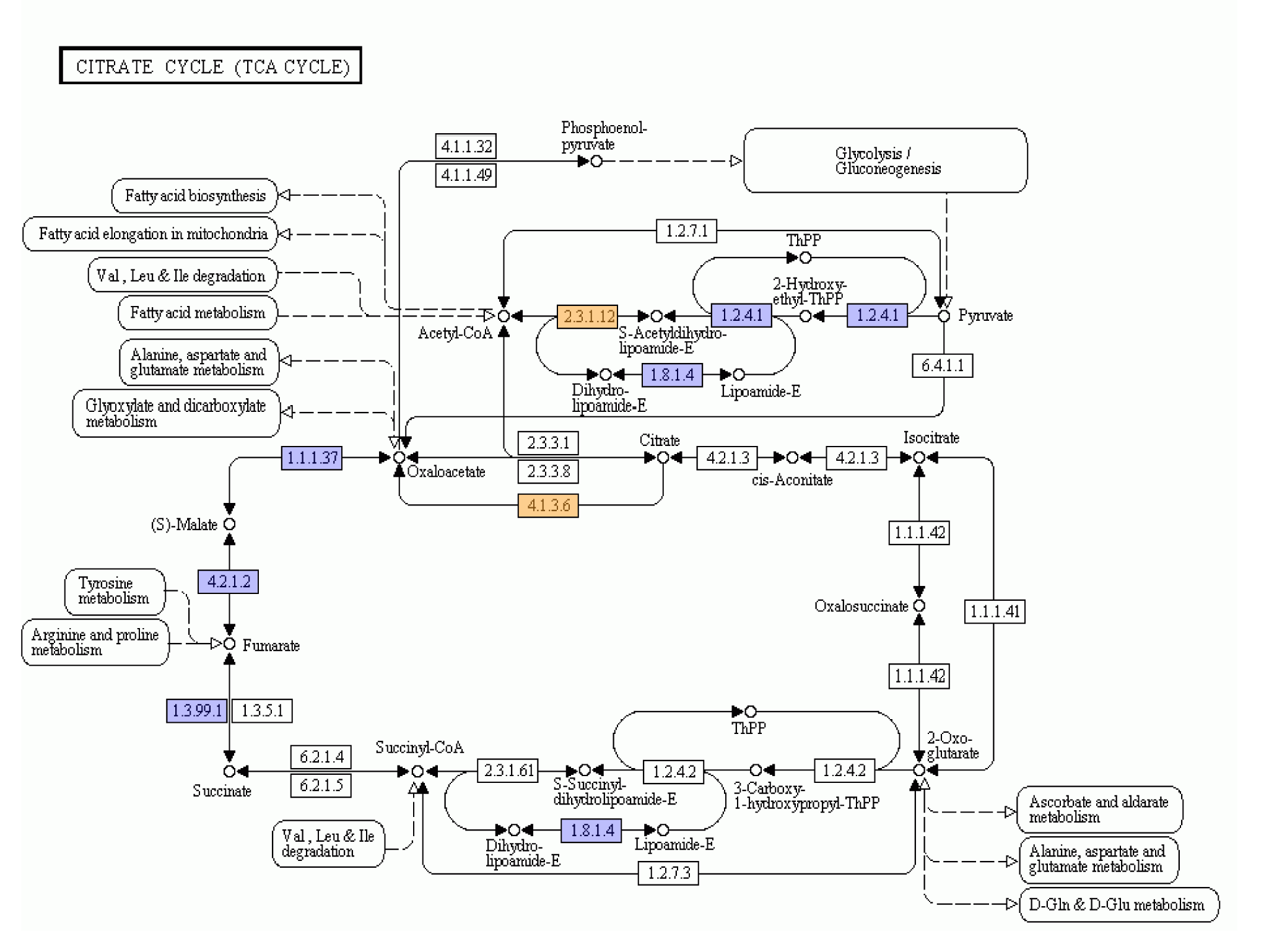

Supplement: Figure S6 — Prediction of additional enzymes tricarboxylic acid (TCA) pathway in L. reuteri ATCC 55730 and partial TCA pathway in L. reuteri ATCC PTA 6475. Genes encoding the enzymes represented in blue are present in both L. reuteri ATCC 55730 and L. reuteri ATCC PTA 6475. Genes encoding the enzymes represented in orange are only present in L. reuteri ATCC 55730. EC:1.1.1.37: malate dehydrogenase; EC:1.2.4.1; pyruvate dehydrogenase (acetyl-transferring); EC:1.3.99.1: succinate dehydrogenase; EC:1.8.1.4: dihydrolipoyl dehydrogenase; EC:2.3.1.12: dihydrolipoyllysine-residue acetyltransferase; EC:4.1.3.6: citrate (pro-3S)-lyase (3 subunits represented by 3 different genes in L. reuteri 55730); EC:4.2.1.2: fumarate hydratase. Figure was obtained by projecting genes present in the TCA pathway in strain 55730 and 6475 via the Integral Microbial Genomes Platform: http://img.jgi.doe.gov. (TIF) [file pone.0018783.s006.tif]

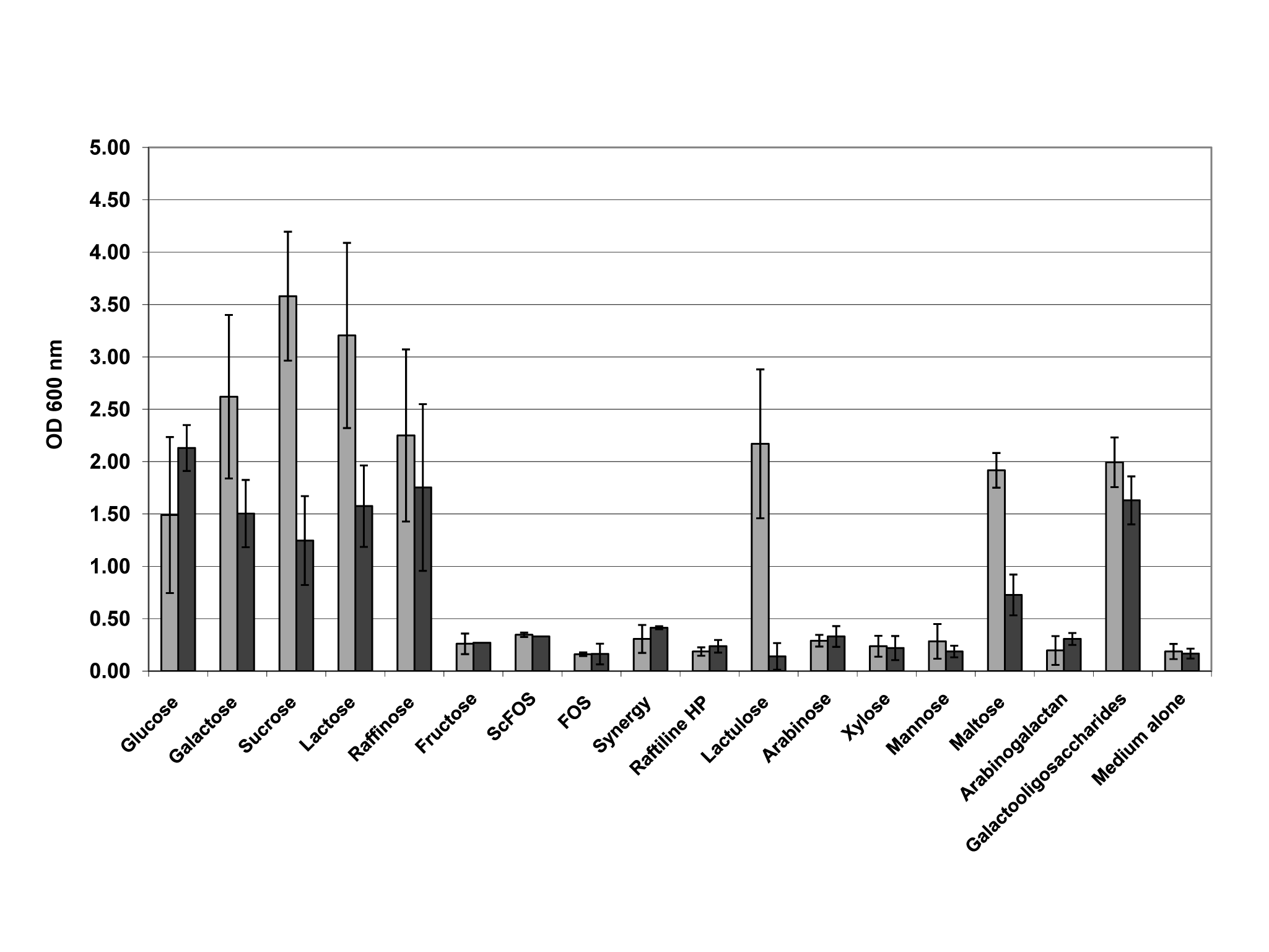

Supplement: Figure S7 — Final OD600 nm reached by L. reuteri ATCC 55730 (light grey) and L. reuteri ATCC PTA 6475 (dark grey) after 24 h of growth in LDM medium with 20 g/L of different carbon source. Strains were grown in anoxic conditions at 37°C. FOS: fructooligosaccharide; ScFOS: fructooligosaccharides; Raftiline HP: long-chain inulin. Error bars represent standard deviations. Data represent the average of 3 biological replicates. (TIF) [file pone.0018783.s007.tif]
